# Plant hyperaccumulators: a state-of-the-art review on mechanism of heavy metal transport and sequestration

**DOI:** 10.3389/fpls.2025.1631378

**Published:** 2025-07-23

**Authors:** Basharat Ahmad Bhat, Muneeb Ahmad Rather, Tanveer Bilal, Romaan Nazir, Roof Ul Qadir, Rakeeb Ahmad Mir

**Affiliations:** ^1^ Department of Bio-Resources, Government Degree College for Women Pulwama, University of, Kashmir, J&K, India; ^2^ Department of Botany, School of Biological Sciences, University of Kashmir, Srinagar, J&K, India; ^3^ Plant Molecular Biology and Biotechnology Lab, Council of Scientific and Industrial Research (CSIR)-Indian Institute of Integrative Medicine, Srinagar, J&K, India; ^4^ Department of Botany, S. P. College, Cluster University, Srinagar, J&K, India; ^5^ Department of Biotechnology, Central University of Kashmir, Ganderbal, J&K, India

**Keywords:** hyperaccumulators, homeostasis, phytoremediation, transporter proteins, metal ion tolerance, microbes

## Abstract

Soils contaminated with heavy metals (HMs) pose severe consequences to living organisms, primarily affecting human health. During the past two decades, researchers have focused on hyperaccumulator plant species to augment the cleanup efforts of contaminated soils. Plants are continually exposed to HMs in the environment since they are sessile organisms. Plants that do not hyperaccumulate metals are vulnerable to high metal concentrations. Their root vacuoles create complexes with metal ligands as a detoxifying approach. On the other hand, metal-hyperaccumulating plants have evolved internal regulatory systems that allow them to hyperaccumulate excess HMs in their above-ground tissues. Unlike metal non-hyperaccumulators, they have the unusual ability to successfully carry out regular physiological activities without displaying any evident stress signs. The capacity of hyperaccumulators to acquire extra metals is due to the overexpression of constitutive metal transporter and their translocation capacity. To accomplish this, plants respond to HMs stress by inducing specifying key genes and enzymes involved in HMs chelation and compartmentalization in plants, such as phytochelatin synthases (*PCS*), which synthesize phytochelatins for metal binding, and metallothionein’s (MTs), which also participate in metal detoxification. Additionally, transporters like ATP-binding cassette (*ABC*) transporters, natural resistance-associated macrophage proteins (*NRAMPs*), and heavy metal ATPases (*HMAs*) facilitate metal sequestration into vacuoles or apoplasts. Genes encoding these proteins (e.g., *PCS1, MT1/2, HMA3/4, and NRAMP3/4*) are often upregulated under heavy metal stress, enabling plants to mitigate toxicity through chelation and compartmentalization. The current review provides an updated overview of major hyperaccumulator plants, explores insights into metal ion transporters and their expression patterns, and discusses the possible molecular mechanisms underlying metal ion hyperaccumulation. In addition, the evolution of various metal ion transporters and their tissue-specific expression patterns have been documented.

## Graphical Abstract

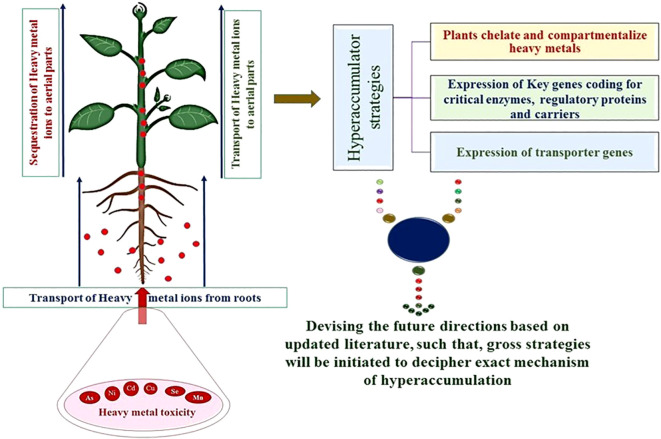

## 1 Introduction

Environmental contamination by HMs and toxic pollutants is a growing global concern, posing severe risks to ecosystems, agriculture, and human health (Briffa et al., 2020; Hembrom et al., 2020). Industrial activities, mining, improper waste disposal, and the excessive use of chemical fertilizers have led to the accumulation of hazardous substances such as cadmium, lead, arsenic, and mercury in soil and water. These contaminants disrupt soil fertility, reduce crop yields, and enter the food chain, leading to chronic diseases in humans and animals (Pirzadah et al., 2019; Dehkordi et al., 2024). Traditional remediation methods, such as chemical treatments and excavation, are often expensive, energy-intensive, and can further degrade the environment. As a result, there is an urgent need for sustainable and cost-effective solutions to detoxify polluted environments (Kuppan et al., 2024; Sangeetha and Jagtap, 2024). HMs are phytotoxic compounds of metals and metalloids, which may be toxic to plants even at low concentrations. While HMs are essential to plants, substantially, there are non-essential HMs like chromium (Cr), cadmium (Cd), lead (Pb), manganese (Mn), etc. induce devastating negative impacts on plant growth, culminating in poor crop production and toxicity to human health (Varma, 2021; Lone and Gaffar, 2021; Varma, 2021; Raza et al., 2022; Bhat et al., 2023). One of the major global concerns regarding animal health and environmental imbalances is the rapid accumulation of HMs to the extent of toxic levels. Substantial loss in crop production is due to the accumulation of heavy metal ions by selective plant hyperaccumulators (Bharwana et al., 2013; Dar et al., 2020; Sharma and Kumar, 2021). Phytotoxic effects of HMs include damage to various physiological and metabolic networks at the cellular and molecular levels (Raza et al., 2022; Bhardwaj et al., 2023). Several metal ions act as potent carcinogens and toxins to animals, usually accumulated in food chains through anthropogenic activities (Arora and Chauhan, 2021; Priyadarshanee et al., 2022; Basu et al., 2023). These HMs in diverse soils may be emitted from coal mines, petrochemical spillages, metal disposals, industrial areas, animal manures, atmospheric depositions, and sewage-sludge treatment plants (Adeyemi, 2021; Oladoye et al., 2021; Tariq et al., 2023). Heavy metal ion toxicity is primarily due to their oxidation ability. In this state, they manifest heavy damage to plants through their negative impacts on physiology, biochemical network, and morpho-anatomy. In addition, HMs inactivate critical enzymes, proteins, and respiratory metabolism and mediate photosynthetic inhibition (Bharwana et al., 2013; Mushtaq et al., 2021b; Thakur et al., 2021). The interaction between hyperaccumulator plants and their environment has far-reaching implications. On one hand, they can improve soil quality by removing toxic elements, making land safer for agriculture (Zhakypbek et al., 2024). On the other hand, their ability to concentrate HMs may affect neighboring plant growth, either by reducing competition (since few plants thrive in metal-rich soils) or by altering microbial communities in the rhizosphere (Barra Caracciolo and Terenzi, 2021; Solomon et al., 2024). For agriculture, hyperaccumulators can be strategically used in phytomining recovering valuable metals like nickel or zinc while also rehabilitating contaminated fields for future crop production. Additionally, their integration into agroecological systems could reduce dependency on chemical remediation, promoting sustainable farming practices (Cao et al., 2025). Understanding these dynamics is essential for optimizing phytoremediation strategies and ensuring their benefits extend to food security, ecosystem restoration, and human well-being.

The hyperaccumulation of metal ions is a highly complex natural phenomenon, mainly due to the expression of unique traits, which are easy to assess. The flexibility of assessing these metal ions largely relies on simple analytical techniques. In addition, hyperaccumulators are of great interest as an alternate strategy to reduce the contamination of soils by toxic metal ions (Pp and Puthur, 2021; Rai et al., 2022; Tariq et al., 2022). Several studies paved the way for opening doors to evolving diverse processes, such as phytomining/bio-fortification, phytoremediation, etc., to improve the efficiency of crops in accumulating nutrients (Clemens et al., 2002; Pirzadah et al., 2015; Yan et al., 2020; Raza et al., 2021). At least 450 species of angiosperms have been identified as potential sinks to hyperaccumulate HMs viz. As, Cu, Cd, Co, Mn, Ni, Pb, Se, Sb, Ti, and Zn. The hyperaccumulation of metal ions is a potential defense against attacks by pathogens or herbivores (Dueli et al., 2021; Mushtaq et al., 2021a; Mir et al., 2022). They employ at least four mechanisms to accumulate metal ions, *viz*. transport of metals through roots from the soil, radial metal ion transport in roots, root to shoot metal accumulation, and detoxification at storage sites (Clemens, 2001; Clemens et al., 2002; Mari and Lebrun, 2005; Merlot et al., 2018; Corso and de la Torre, 2020). Almost all hyperaccumulators have genetically adapted to accumulate various metal ions and have been used in phytomining technologies for their extraction (van der Ent et al., 2015; Yaqoob et al., 2023; Bhat et al., 2024). Metal homeostasis and stress tolerance are linked to understanding the hyperaccumulation mechanism of hyperaccumulators. In addition, these plant species have evolved to possess adaptations for hypertolerance and detoxification of metal and metalloids (Angulo-Bejarano et al., 2021; Peng et al., 2021; Rai et al., 2021).

Adapting hyperaccumulators to survive under extreme metal ion concentration may further facilitate understanding molecular mechanisms to detoxify the soils. Unraveling the molecular basis of the hyperaccumulation mechanism helps develop proper phytoremediation and phytoextraction techniques. Higher biomass and enhanced growth of roots and shoots may further be augmented by employing ideal hyperaccumulators through phytoremediation (Malik et al., 2015; Quarshie et al., 2021; Ali et al., 2022; Ojuederie et al., 2022). In addition, targeting contaminated soils by specific hyperaccumulators will further enhance crop production and homeostasis of metal ions. The traits possessed by hyperaccumulators serve two essential aspects of the ecosystem: phytoremediation and the other is the biofortification of metal ions (Pirzadah et al., 2019; Jiang et al., 2021; Yang et al., 2021; Pirzadah et al., 2022). In other words, hyperaccumulators accumulate a particular metal ion a hundred or thousand times more than the normal concentration accumulated by common plants. In addition, they also can detoxify these metal ions to maintain their growth and metabolism. Only a few plant species accumulate large amounts of metalloids or transition metal ions like Zn, Cd, Ni, Se, As, Cu, Pb, Mn, Tl, Co, or Sb in their aerial part at higher concentrations as compared to other plant species. Reeves et al. (2018) reported several hyperaccumulators of metal ions, such as Ni (532 species), followed by Cd (07 species) and (05 species).

This review summarizes the molecular mechanism behind the transport and sequestration of metal ions such as Ni, Cu, Zn, Cd, Mn, As, and Se through the intervention of hyperaccumulators. In addition, updated information regarding the expression pattern of transporter genes is provided.

## 2 Heavy metal ion hyperaccumulators-an update

Hyperaccumulators are plant species that accumulate metal ions at high concentrations from contaminated soils in xylem from roots to shoots through bulk flow. The prominent families of plants belonging to the hyperaccumulator category include families such as *Asteraceae, Brassicaceae, Buxaceae, Cunoniaceae, Euphorbiaceae, Flacourtiaceae, Phyllanthaceae, Rubiaceae, Salicaceae*, and *Violaceae* (Reeves, 2000; Krämer, 2010; Reeves et al., 2018; Zhang et al., 2021). Numerous metal ion hyperaccumulators have been identified (**Table 1**), possessing great potential to be employed in phytoextraction and phytoremediation techniques.

**Table 1:** Major hyperaccumulators and metal ions transported.

S. No.	Species	Name of metal ion transported	References
1.	*Arabidopsis halleri*	Zn, Cd and Fe	(Hanikenne et al., 2008; Assunção et al., 2010)
2.	*T. caerulescens*	Zn, Fe, Cd and Ni	(Assunção et al., 2010)
3.	*T. goesingense*	Zn and Ni	(Persans et al., 2001)
4.	*Convolvulus arvensis* L.	Cu, Fe, Mn, and Zn	(Massa et al., 2010)
5.	*Ranunculus sceleratus L.*	Manganese *(Mn)*	(Farahat and Galal, 2018)
6.	*Taraxacum officinale*	Zn and Fe	(Massa et al., 2010)
7.	*Carduus* nutans L.	Cd (Cadmium)	(Palutoglu et al., 2018)
8.	*Lantana camara* L.	Cd (Cadmium)	(Liu et al., 2019)
9.	*S. plumbizincicola*	Cd (Cadmium)	(Gendre et al., 2007)
10.	*Sedum alfredii*	Cd (Cadmium), Zinc (Zn)	(Deng et al., 2007)
11.	*Phragmites australis*	Cu (Copper)	(Ashraf et al., 2011)
12.	*Typha latifolia* L.	Cu (Copper)	(Anning and Akoto, 2018)
13.	*N. nucifera*	Cu (Copper)	(Ashraf et al., 2011)
14.	*Stachys inflata* Benth.	Cu (Copper)	(Mohsenzadeh and Mohammadzadeh, 2018)
15.	*Sedum alfredii* Hance	Zn (Zinc)	(Deng et al., 2007; Krämer, 2010; Kozhevnikova et al., 2017)
16.	*Brassica napus* L.	Zn (Zinc)	(Belouchrani et al., 2016)
17.	*Armeria maritima* subsp*. halleri*	Zn (Zinc)	(Bothe, 2011)
18.	*Viola lutea* subsp*. calaminaria*	Zn (Zinc)	(Bothe, 2011)
19.	*Salvinia molesta* D. Mitch.	Lead (Pb)	(Ashraf et al., 2011)
20.	*Noccaea caerulescens*	Cadmium (Cd)	(Deng et al., 2007; Moameri et al., 2017)
21.	*Malva pusilla* Sm.	Cadmium (Cd)	(Wu et al., 2018)
22.	*Lactuca orientalis* Boiss.	Chromium (Cr)	(Antoniadis et al., 2017)
23.	*Tragopogon collinus* DC.	Chromium (Cr)	(Antoniadis et al., 2017)
24.	*Brassica juncea* (L.) Czern.	Chromium (Cr)	(Antoniadis et al., 2017)
25.	*Cichorium* sp*inosum* L.	Chromium (Cr)	(Antoniadis et al., 2017)
26.	*Stipa hohenackeriana*	Nickel (Ni)	(Bani et al., 2015; Drozdova et al., 2017; Moameri et al., 2017)
27.	*Odontarrhena muralis*	Nickel (Ni)	(Bani et al., 2015; Drozdova et al., 2017; Moameri et al., 2017)
28.	*Silene vulgaris*	Nickel (Ni)	(Bani et al., 2015; Drozdova et al., 2017; Moameri et al., 2017)
29.	*Arabidopsis thaliana*L.	Palladium (Pd)	(Harumain et al., 2017)
30.	*Scopelophila ligulata*	Iron (Fe)	(de la Fuente et al., 2017; Nakajima and Itoh, 2017)
31.	*Cyperus rotundus* L.	Tin	(Ashraf et al., 2011)
32.	*Melastoma malabathricum* L.	Tin	(Ashraf et al., 2011)
33.	*Alternanthera bettzickiana*	Lead (Pb)	(Tauqeer et al., 2016)
34.	*Cortaderia hapalotricha*	Lead (Pb)	(Bech et al., 2016)
35.	*Thlaspi arvense L.*	Nickel (Ni) and Zinc (Zn)	(Reeves and Brooks, 1983)
36.	*Prunus cerasifera Ehrh.*	Cu and Ni	(Turkyilmaz et al., 2018)
37.	*Tilia tomentosa Moench*	Cr, Cd and Pb	(Turkyilmaz et al., 2018)
38.	*Salix schwerinii*	Chromium (Cr), Copper (Cu) and Zinc (Zn)	(Salam et al., 2016)
39.	*Elaeagnus angustifolia L.*	Cu, Ca, Cd, Cr, Fe, Mg, Mn, Ni, Pb, and Zn	(Turkyilmaz et al., 2018)
40.	*Pteris vittata L.*	Arsenic (As) and Lead (Pb)	(Ashraf et al., 2011; Wan et al., 2017)
41.	*Aesculus hippocastanum L.*	Cu, Ca, Cd, Cr, Fe, Mg, Mn, Ni, Pb, and Zinc (Zn)	(Turkyilmaz et al., 2018)
42.	*Betula pendula Roth*	Cu, Ca, Cd, Cr, Fe, Mg, Mn, Ni, Pb, and Zinc (Zn)	(Turkyilmaz et al., 2018)
43.	*Platanus orientalis L.*	Cu, Ca, Cd, Cr, Fe, Mg, Mn, Ni, Pb, and Zinc (Zn)	(Turkyilmaz et al., 2018)
44.	*Eleocharis acicularis (L.)*	Ag, Cu, Cd, Pb, and Zn	(Ha et al., 2011)
45.	*Imperata cylindrica*	Lead (Pb), Copper (Cu), Tin	(Ashraf et al., 2011)
46.	*Conium maculatum L.*	Lead (Pb) and Zinc (Zn)	(Mohsenzadeh and Mohammadzadeh, 2018)
47.	*Molinia caerulea (L.)*	Cadmium (Cd), Lead (Pb) and Zinc (Zn)	(Pietrzykowski et al., 2018)
48.	*Salix viminalis L.*	Lead (Pb) and Zinc (Zn)	(Mleczek et al., 2018)
49.	*Arabidopsis halleri*	Zinc (Zn) and Cd (Cadmium)	(Hanikenne et al., 2008)
50.	*Arabidopsis lyrata*	Zinc (Zn) and Cd (Cadmium)	(Hanikenne et al., 2008)
51.	*Justicia procumbens*	Zinc (Zn)	(Reeves et al., 2018)
52.	*N. caerulescens*	Zinc (Zn) and Cadmium (Cd)	(Papoyan et al., 2007; Halimaa et al., 2019)

In addition, several plants, such as *Brassicaceae*, *Noccaea caerulescens*, *Arabidopsis thaliana, Chicorium* sp*inosum, and Sedum alfredii.* Hance, and *Silene vulgaris O. muralis*, are studied in detail to have deep insights into understanding the mechanism of hyperaccumulation (Kaushal et al., 2021; Zhang et al., 2021). Several hyperaccumulators, such as *Aesculus hippocastanum* L., *Betula pendula* Roth, *Elaeagnus angustifolia* L., *Fraxinus excelsior* L., *Platanus orientalis* L., and *Tilia tomentosa* Moench, have been employed in biomonitoring of metal ions, such as Cd, Cr, Cu, Ca, Fe, Mg, Mn, Ni, Pb, and Zn (Turkyilmaz et al., 2018). It is reported that various populations of *N. caerulescens* vary in their hyperaccumulation of metal ions, such as; Zn, Ni, and Cd (Assunção et al., 2003; Manara et al., 2020; Sytar et al., 2020; Tariq et al., 2021). Studies reveal that transporter genes and proteins expressed by hyperaccumulators are highly efficient in contributing to metal tolerance and detoxification of HMs (Sharma et al., 2021b; Bhat et al., 2022b). Since, HMs have the least mobility in soils, plants must adopt diverse mechanisms to transport metal ions efficiently. The underlying molecular mechanisms of heavy metal ion hyperaccumulation are unraveled by employing molecular and genetic systems of hyperaccumulators.

Furthermore, Arabidopsis CPx P1B-type ATPases such as *HMA3* (engaged in lead storage) and *HMA4* (involved in lead transport) translocate this metal across biological membranes in an energetically-driven process (Gupta et al., 2013). The fact that lead competes with calcium in this transport system explains why lead inhibits voltage-gated Ca-channels (Kumar et al., 2017). As a defensive strategy, phytochelatin complexation sequesters lead into vacuoles via vascular flow, while the remaining lead is transferred through the xylem, and the apoplast is translocated to the leaf.

### 2.1 Tissue-specific hyperaccumulation

Metal ion hyperaccumulation is tissue/organ-specific depending on the type of species and the transporters (Pasricha et al., 2021; van der Ent et al., 2021). For example, in a comparative study, it was observed that *A. maritima* subsp. *halleri* accumulated 88- and 20-times Cu and Pd in roots, respectively, compared to leaves. In addition, experiments also reveal that Pb, Cd, Zn, and Cu were found 3 to 8 times more in brown leaves than green leaves of *A. maritima* subsp*. halleri* (Dahmani-Muller et al., 2000). In *A. halleri*, differential accumulation of Zn (>20,000 mg kg^-1^) and Cd (>100 mg kg^-1^) was observed in leaves rather than in other aerial tissues (Dahmani-Muller et al., 2000). *Lantana camara* L., a native plant of America and Africa, accumulated>100 mg kg^-1^ of Cd in its shoots (Liu et al., 2019). Moameri et al. (2017) reported that Cd hyperaccumulated up to 68.47 mg kg^-1^ in *Lactuca orientalis* and up to 68.1 mg kg^-1^ in roots of *T. collinus* and 62 mg kg^-1^ in both shoots and roots of *B. juncea* (Moameri et al., 2017). Scanning and transmission electron microscopy coupled with energy-dispersive X-ray (SEM and TEM with EDX), histochemical staining, inductively coupled plasma mass spectrometry (ICP-MS), and optical microscopy (OM) revealed that *Imperata cylindrica* (L.) P. Beauv hyperaccumulates iron (Fe) in the intercellular spaces of aerial tissues (de la Fuente et al., 2017). Cadmium is accumulated in the edges of leaves, epidermal cells, cell walls, and metabolically less active parts of leaves in *Noccaea caerulescens* (Cosio et al., 2005). In addition, Cd is also accumulated in mesophyll cells of leaves in *A. halleri* (Küpper and Kochian, 2010).

### 2.2 Nickel hyperaccumulators

Nickel (Ni) belongs to the essential category of metal ions, but its serious negative consequences appear when its concentration in plants exceeds 0.85 mM kg^−1^ plant dry biomass (Rosatto et al., 2021). The Ni toxicity leads to inhibitory effects on the enzymes necessary for operating the Calvin cycle and chlorophyll biosynthesis. It may produce reactive oxygen species (ROS) (Sachan and Lal, 2017). Several plant species have adapted mechanisms for hyperaccumulation and detoxification to circumvent Ni toxicity. Global hyperaccumulator databases have documented 721 metal ion hyperaccumulators, among which Ni is hyperaccumulated by about 532 plant species (Reeves et al., 2018). Most hyperaccumulators (340 plant species) belong to only five families having 180 species to *Phyllanthaceae*, 87 species to *Brassicaceae*, 48 species to *Cunoniaceae*, and 42 species to *Euphorbiaceae*. Recent Ni hyperaccumulator additions include *Senecio conrathii* and *Phyllanthus rufuschaneyi* (Bouman et al., 2018; Siebert et al., 2018). Ni hyperaccumulators are majorly found in serpentine soils, including *Alyssum sibiricum* and *Senecio coronatus* (Reeves and Adigüzel, 2004; Boyd et al., 2008) and *C. bursa-pastoris* (Seregin and Kozhevnikova, 2006). Ni hyperaccumulators outnumber among plant species *viz.-a-viz*. other metal ions (Krämer, 2010; Reeves et al., 2018). Numerous hyperaccumulators, such as *Berkheya coddii* Roessler, *Echium amoenum* Fisch. & C.A. Mey, *Stipa hohenackeriana, Lens orientalis*, and *Taeniatherum crinitum* (Schreb.) Nevski have also been identified for Ni accumulation (Robinson et al., 2003; Moameri et al., 2017). Several species of ferns, liverworts, and mosses hyperaccumulate Ni from their habitats (Seregin and Kozhevnikova, 2021). *O. muralis* has been identified as a hyperaccumulator of Ni using X-ray diffraction (XRD), gravimetric analysis, and inductively coupled plasma atomic emission spectroscopy (ICP-AES) (Zhang et al., 2016). In addition, the *Brassicaceae* family has been reported to accumulate three times more Zn and six times more Ni than other hyperaccumulators (Krämer, 2010).

Angiosperms predominantly accumulate Ni, as evident in the reports of 140 species grown on the islands of New Caledonia and Cuba (Whiting et al., 2004; Jaffré et al., 2013). Hyperaccumulators like *Alyssum bertolonii* and *Hybanthus floribundus* were reported to hyperaccumulate Ni (Minguzzi, 1948; Severne and Brooks, 1972). The *Pycnandra* (previously *Sebertia*) *acuminate* accumulates 2 to 3 orders of Ni in their shoots compared to non-accumulator plant species (Jaffré et al., 1976). Drozdova et al. (2017) also reported that *O. muralis* hyperaccumulates Ni in their aerial parts. Moreover, it is said that *S. hohenackeriana* hyperaccumulates Ni in roots up to 195 mg kg^-1^ and shoots up to 119 mg kg^-1^ (Moameri et al., 2017). Differential hyperaccumulation rate of Ni was observed in plants; for example, *E. amoenum* accumulates up to 21 mg kg^-1^ of Ni in roots and 57 mg kg^-1^ in shoots, whereas, *L. orientalis* accumulates up to 32 mg kg^-1^ Ni in roots and 36 mg kg^-1^ in shoots (Moameri et al., 2017). The results of almost similar accumulation rates are displayed by *T. crinitum*, with an accumulation rate of 40 mg kg^-1^ of Ni in roots and 34 mg kg^-1^ in shoots (Moameri et al., 2017). Likewise, *S. hohenackeriana* hyperaccumulates up to 54 mg kg^-1^ of Ni in shoots and 59 mg kg^-1^ in roots (Moameri et al., 2017). The available hyperaccumulator database may serve as the baseline for employing the remediation measures to decontaminate Ni toxicity from agricultural soils.

### 2.3 Zinc hyperaccumulators

In general, zinc (Zn) is classified as an essential micronutrient due to its direct physiological and metabolic significance in plants. However, when present in concentrations exceeding the threshold level (>10,000 mg/kg), zinc becomes toxic, leading to impaired growth, disrupted physiological functions, and even plant death. At such elevated levels, zinc exerts several inhibitory effects, particularly on the photosynthetic metabolism and overall growth processes of crops (Ali et al., 2020; Bhat et al., 2020; Angulo-Bejarano et al., 2021; Kaur and Garg, 2021). Many plant species accumulate Zn at concentrations of up to 1% of the dry weight of plant biomass (Noulas et al., 2018; Sytar et al., 2021). At least nine species of Zn hyperaccumulators belong to the *Brassicaceae* family, most of which have been found in contaminated soils. Zn is also hyperaccumulated by *L. ruderale, C. bursa*-*pastoris*, and *A. halleri* (Küpper et al., 2000; Kozhevnikova et al., 2017; Raza et al., 2020). Interestingly, numerous species belonging to the family *Brassicaceae* accumulate multiple heavy metal ion (Dar et al., 2018). Ni is accumulated at 1000–30000 μg g^-1^ of dry mass basis, whereas Zn up to 1000 μg g^-1^ of dry mass basis) *Thlaspi* species belonging to *Cruciferae* (mustard family) were collected from Europe (Reeves and Brooks, 1983). Few populations of *N. caerulescens* show minor symptoms upon accumulating Cd up to 4000 mg kg^-1^ dry weight and 30,000–40,000 mg kg^-1^ dry weight of Zn (Shen et al., 1997; Ebbs et al., 2002; Natasha et al., 2022). Zn is hyper-accumulated by *A. halleri* compared to facultative accumulation of Pb and Cd (Bert et al., 2000; Küpper et al., 2000; Bert et al., 2002; Pauwels et al., 2006; Stein et al., 2017). In addition, *A. halleri* shows species-wide Zn and Cd hypertolerance, with significant variation among its various populations (Pauwels et al., 2006; Meyer et al., 2010; Corso et al., 2018). It is evident from the above findings that a more significant amount of metal ions is accumulated in aerial parts of the hyperaccumulators. Zn and Cd are hyperaccumulated by almost all the subspecies and populations of *Arabidopsis halleri* found in the soils of both contaminated and non-contaminated habitats (Bert et al., 2002). Few populations of *N. caerulescens* found in southern France accumulate up to 2908 μg g^−1^ of Cd in their leaves (Reeves et al., 2001). In addition, many *N. caerulescens* populations hyperaccumulate Ni and Zn from ultramafic soils (Reeves et al., 2001). Significant variation in Cd, Ni, and Zn accumulation has been reported in different populations of *N. caerulescens* (Lloyd-Thomas, 1995; Reeves et al., 2001). Hydroponic experiments reveal that varying concentrations of Cd and Zn are hyperaccumulated by three species of *Sedum alfredii* (Deng et al., 2007; Li et al., 2007). These reports will pave the way to understanding the physiological process modulated by the Zn accumulation in cellular compartments and tissues in a specific crop. In addition, one can employ newer technology like genome editing to understand the mechanism of heavy metal ion transport and sequestration in plants (Riyazuddin et al., 2022; Venegas-Rioseco et al., 2022).

### 2.4 Mercury hyperaccumulators

In plants, does not play any physiological role (Rascio and Navari-Izzo, 2011). It is adsorbed from the soil as a soluble complex and precipitated as phosphate, carbonate, sulphide, and hydroxide (Tangahu et al., 2011). Phytotoxic effects of high mercury levels on plants are possible (Azevedo and Rodriguez, 2012; Rocha et al., 2019). It affects oxidative metabolism and photosynthesis by interfering with the electron transport mechanism in mitochondria and chloroplast. This induces cell disruption by causing the creation of ROS. Hg is also responsible for limiting aquaporin activity and lowering plant water absorption. The permeability of cell membranes and the number of palisades may be reduced in the presence of Hg, resulting in the buildup of Fe and the loss of essential elements like Mgand K (Shiyab et al., 2009; Tangahu et al., 2011; Azevedo and Rodriguez, 2012). Hg interaction with thiol (SH) groups in tissues rich in SH ligands, such as seed and embryo, results in the development of an S-Hg-S bridge, which disrupts the group’s stability. Seed germination and embryo development are both affected by this binding. Hg also affects the antioxidant defense system by altering enzymatic and non-enzymatic antioxidants and disrupting cells (Azevedo and Rodriguez, 2012), negatively affecting light and dark photosynthetic responses. Photosynthesis is disrupted when Hg replaces the central Mg atom in chlorophyll (Muddarisna et al., 2013; Zhao et al., 2014). Although it is mostly stored in roots, it can accumulate in tiny amounts in shoots by translocating soluble forms or directly absorbing the vapor form (Ranieri et al., 2021). The plant absorbs ionic, methyl, and phenyl forms of mercury from the soil. The phenyl form is used for absorption, whereas the methyl form is used for sequestration. The change of phenyl mercury to methyl mercury is high in apical regions, whereas the transformation of phenyl mercury to ionic mercury is strong in subtending internode regions (Gay and Butler, 1977).

### 2.5 Cadmium hyperaccumulators

Cadmium (Cd) is another metal ion imparting high toxicity with high transport mobility to the plant. It is found to cause extensive damage to metabolic networks (Liao et al., 2015; Liu et al., 2022). Cd is hyperaccumulated by *A. halleri*, *A. halleri* ssp. *Gemmifera*, and *A. lyrata* (Huguet et al., 2012; Isaure et al., 2015; Fukuda et al., 2020). Cd is a non-essential element usually found in hyperaccumulators’ roots and aerial organs (Conn and Gilliham, 2010; Imperiale et al., 2022). Several hyperaccumulator species have accumulated Cd, including *A. halleri*, *B. juncea, Lactuca orientalis* Boiss., *N. caerulescens, Tragopogon collinus* DC., and *S. hohenackeriana* (Cosio et al., 2005; Küpper and Kochian, 2010; Moameri et al., 2017). Hydroponics-based experiments reveal that *Noccaea caerulescens* was more tolerant to Cd grown under high zinc concentration and accumulated higher concentrations of Cd/Zn (Papoyan et al., 2007). Through biomonitoring analysis of (HMs), it was observed that Ni and Cu were hyperaccumulated by *Prunus cerasifera* Ehrh., whereas Pb and Cd were effectively accumulated by *T. tomentose* (Turkyilmaz et al., 2018). Analysis based on Atomic Absorption Spectroscopy (AAS) showed that *T. latifolia* hyperaccumulated Cu and Cd, while *E. crassipes* accumulated Pb when grown in wetlands supplied with effluents (Sukumaran, 2013). It has been reported that Iron (Fe) was hyperaccumulated by *Scopelophila ligulata* (Spruce) 10 to 61 times more than normal mosses (Nakajima and Itoh, 2017). The hyperaccumulators *Lactuca orientalis, T. collinus*, and *B. juncea* accumulates Cd in roots and shoots (Moameri et al., 2017). Cd hyperaccumulation and tolerance are modulated by overexpression of NRAMPs transporters in *N. caerulescens* (Oomen et al., 2009; Wei et al., 2009; Takahashi et al., 2011). Efficient remediation of Cd from the agricultural and urban soils is critical for sustainable agriculture development in current food insecurity trends. An update on mechanism of hyperaccumulators to initiate phytoremediation of Cd provides efficient ways to restore the polluted habitats to healthy state since it is evident from the existing literature that Cd has negative impacts on the metabolic and physiological networks of plants.

### 2.6 Manganese hyperaccumulators

Manganese (Mn) is an essential category of micronutrients, although in certain climatic-cum-edaphic conditions, primarily in acidic soils, it is toxic to crops (Rashed et al., 2019). It is believed that Mn adversely affects photosynthetic metabolism and enhances ROS generation (Cui et al., 2021). Numerous Mn hyperaccumulator plant species belonging to various families such as *Araliaceae, Apocynaceae, Celastraceae, Clusiaceae, Myrtaceae, Polygonaceae, Proteaceae*, and *Theaceae* have been identified (Fernando et al., 2013). The deposition of higher concentrations of Mn in the vacuoles of photosynthetic cells of the upper epidermis was reported in *Maytenus fournieri* L (Doncheva et al., 2009; Fernando et al., 2012). In addition, in *Gossia*. *Amplexicaulis* L. Mn was deposited in entire leaves, whereas in *Trapa natans* L and *Gossia hillii* L., Mn was hyperaccumulated in the floating lamina and photosynthetic tissues, respectively (Fernando et al., 2012, 2013). Moreover, trichomes of *Alyssum murale* L. and *Helianthus annus* L. also hyperaccumulate Mn (Blamey et al., 1986; Broadhurst et al., 2004).

### 2.7 Lead hyperaccumulators

Lead (Pb) is a non-essential heavy metal with little understanding of its biological use in plants due to its high toxicity. Pb poses significant health threats even at low doses, particularly in child brain development and renal failure (Gaur et al., 2014; Kumar et al., 2017). Lead phytotoxicity inhibits metabolic processes by interfering with enzymes, affecting root elongation, seed germination, and plant development. A high quantity of lead affects chlorophyll and ATP synthesis, cell membrane permeability, water, nutrient intake, seedling growth, and biomass output. Lead poisoning causes oxidative stress-mediated by ROS, which causes protein oxidation, lipid, nucleic acid peroxidation, and eventually death (Nagajyoti et al., 2010; Dewanjee et al., 2015; Li et al., 2016). This is supported by the fact that lead poisoning increases the catalytic activity of antioxidant enzymes (Li et al., 2016). Due to its sorption with soil, lead is not readily accessible in biological systems and has a limited solubility at normal pH, making it unavailable for plant absorption even by hyperaccumulators (Chen et al., 2004; Bahraminia et al., 2016). Some recognized lead hyperaccumulators, such as *Brassica napus* and *Euphorbia cheiradenia*, have been shown to collect more than 1000 mg kg^-1^ of lead in dry weight (Major, 2010; Ali et al., 2013). The apoplastic route or Ca^2+^ channels are both involved in absorbing lead by roots. Alternative mechanisms for lead absorption by roots include cyclic nucleotide-gated ion channels and cation transporters. Lead follows the apoplastic route after absorption by roots, although its transport beyond endodermis is limited by the Casparian strip owing to phytochelatin binding. Sequestration in root vacuoles after complex formation, accumulation in plasma membranes, and complexation with phytochelatins, glutathions, and amino acids like proline all limit lead translocation. By establishing a metal-ligand combination, lead immobilisztion can also occur in the form of phosphates, resulting in reduced negative effects and greater phytoextraction (Kumar et al., 2017). Ethylenediamine tetraacetic acid (EDTA), nitrilotriacetic acid (NTA), and malate are chelating chemicals that can be used to immobilize HM ions (Tangahu et al., 2011; Gaur et al., 2014). Rhizofiltration is when the lead is absorbed and deposited in the roots, with only a small quantity being translocated to the aerial sections of plants like *Typha domingensis* (Bindu et al., 2010).

### 2.8 Selenium hyperaccumulators

Selenium (Se) is another potentially toxic heavy metal ion distributed in trace amounts in the earth’s crust in the form of metalloids (Lima et al., 2018; Reynolds et al., 2020). Toxicity mediated by Se above threshold level exhibits several pathological conditions in plants, such as stunted growth, withering, drying of leaves, reduced protein synthesis, and chlorosis (Mengel and Kirkby, 1987; Van Hoewyk, 2013; Gupta et al., 2022). Several plant species such as *Xylohiza* and *Conopis* hyperaccumulate Se >1000 mg Se kg^-1^ dry weight if grown in Se-rich soils. Two important hyperaccumulators of Se identified are *Astragalus bisulcatus* (Hook.) A. Gray and *Stanleya pinnata* (Pursh) Britton, accumulating Se in reproductive organs and young growing leaves (Freeman et al., 2006). On a side note, (Antoniadis et al., 2017), identified *C.* sp*inosum*, a wild edible vegetable, as a hyperaccumulator of chromium (Cr).

### 2.9 Arsenic hyperaccumulators

Arsenic (As) is a non-essential heavy metal ion highly toxic to crops. It is transported to its roots through specific transporters (Sytar et al., 2021). Hyperaccumulators accumulate about 2% of As in aerial tissues of plants (Chen et al., 2021). More than 21 hyperaccumulators of Arsenic (As) have been identified, and most of them belong to the genus *Pteridaceae* (Xie et al., 2009). *Pteris vittata* (Chinese Brake fern) was identified as a potential hyperaccumulator of As grown at four mining sites in Hunan Province of China (Wan et al., 2017). In addition, *Pteris vittata* (Chinese Brake fern) used phytate, a root exudate, to enhance the uptake of As and increase plant growth and development (Liu et al., 2017). Similarly, two species of *Brassicaceae* also hyperaccumulate arsenic (As) (Karimi et al., 2009). In submerged plants, *Callitriche stagnalis* and *Myriophyllum propinquum*, As is accumulated at 1000 mg kg^–1^ dry weight (Robinson et al., 2006). *Eriophorum angustifolium* hyperaccumulates As in root tissues, while in *Wolffia globose*, up to 400 mg kg^–1^ of As is accumulated (Stoltz and Greger, 2002; Zhang et al., 2009). Certain gymnosperms, such as *Pseudotsuga menziesie*, hyperaccumulate As in needles and stems (Haug et al., 2004).

## 3 Hyperaccumulators as a prelude to solving the heavy metal toxicity: an overview

The molecular mechanism of hyperaccumulation has been primarily based on physiological adaptations by hyperaccumulators, such as increased metal ion uptake, loading in the xylem, and detoxification in aerial parts of the plant (Pasricha et al., 2021). Hyperaccumulators display variable mechanisms to accumulate the metal ions from the contaminated and normal soils. The uptake of excess accumulated metal ions is modified or stored to tolerate their ill effects on the growth and metabolism of hyperaccumulators (Pasricha et al., 2021; Sytar et al., 2021). Moreover, bacteria and fungi generally occur in bound form and are converted into a simple form. In addition, several chelating agents secreted in the rhizosphere further help in the absorption of metal ions by several plasma-bound proteins and for specific metal, ion reductases to facilitate their transportation into aerial parts of plants through xylem (Dotaniya et al., 2015). Several plant species are hyperaccumulators of economically essential metal ions and display considerable tolerance to specific classes of metal ions (Pasricha et al., 2021; Sytar et al., 2021; Tariq et al., 2022).

**Figure 1:**
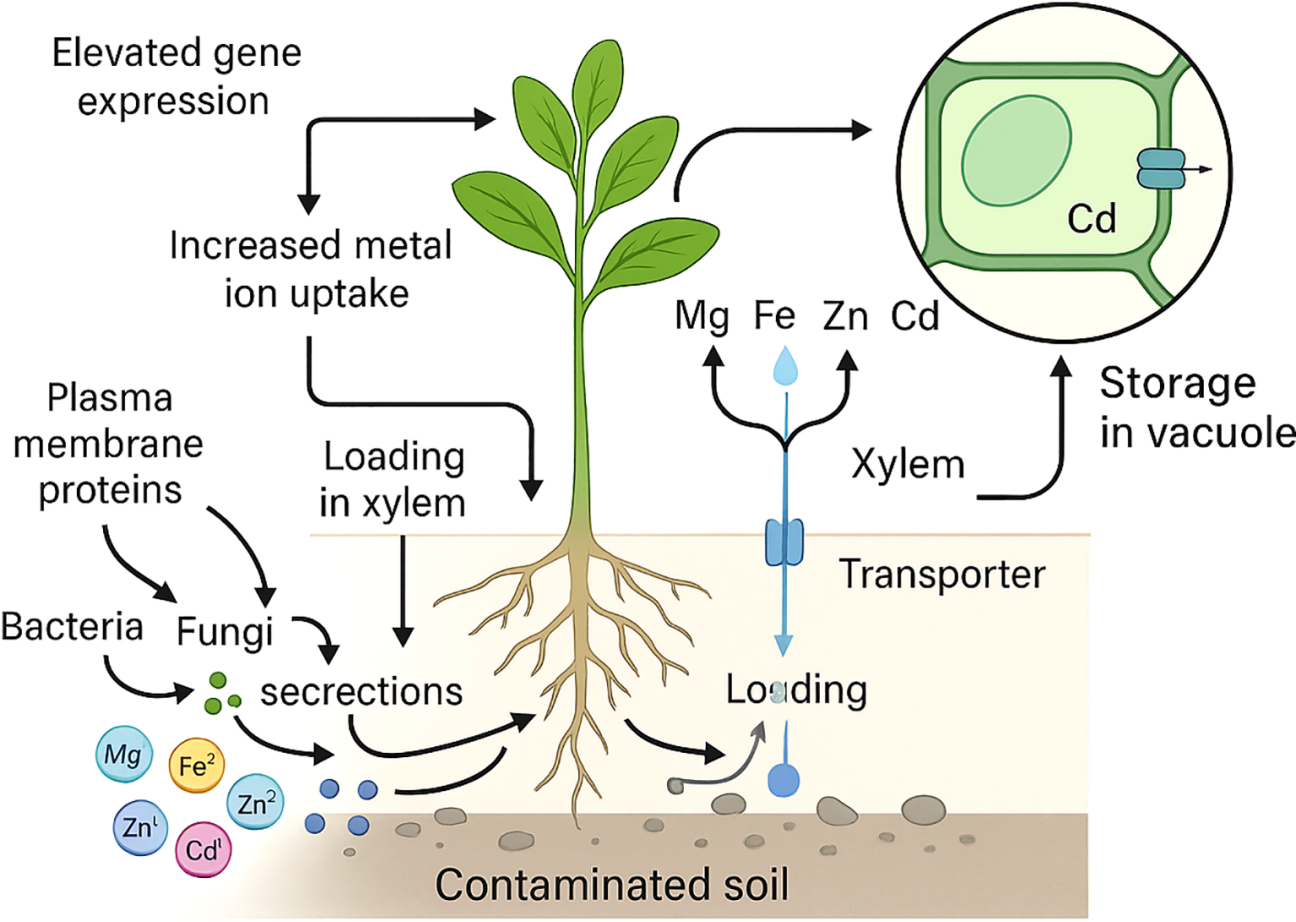
Hyperaccumulators as a prelude to solving the heavy metal toxicity.

Elevated expression of genes coding for transporters and proteins for chelation plays a critical role in hypertoleranace and hyper-accumulation in several plant hyperaccumulators. Several studies have reported the foliar heavy metal concentration in hyperaccumulators (Krämer, 2010; Goolsby and Mason, 2015; Bhat et al., 2022a). The *Alyssum bertolonii*/*Brassicaceae* (Minguzzi, 1948) was first reported to hyperaccumulate Ni, whereas, *Noccaea caerulescens* (formerly, *Thlaspi caerulescens*)/*Brassicaceae* was reported to hyperaccumulate Zn (Sachs, 1865; Baumann, 1885; Reeves, 2000). These reports attracted scientific communities in the early 1990s to employ hyperaccumulators as alternative strategies to circumvent HM toxicity issues. These plant species evolved our mechanistic understanding of molecular mechanisms associated with hyperaccumulation and strategy to detoxify the metal ions. At least tree Quantitative trait locus (QTLs) possibly belonging to hypertolerance of Cd and Zn have been mapped (Courbot et al., 2007; Willems et al., 2007). Moreover, an overlapping QTL identified in the *AhHMA4* (Heavy Metal ATPase 4) gene in *Arabidopsis halleri*, a hyperaccumulator of Cd and Zn, is also screened (Courbot et al., 2007; Willems et al., 2007) (**Figure 1**).

### 3.1 Expression pattern of metal ion transporters

**Figure 2:**
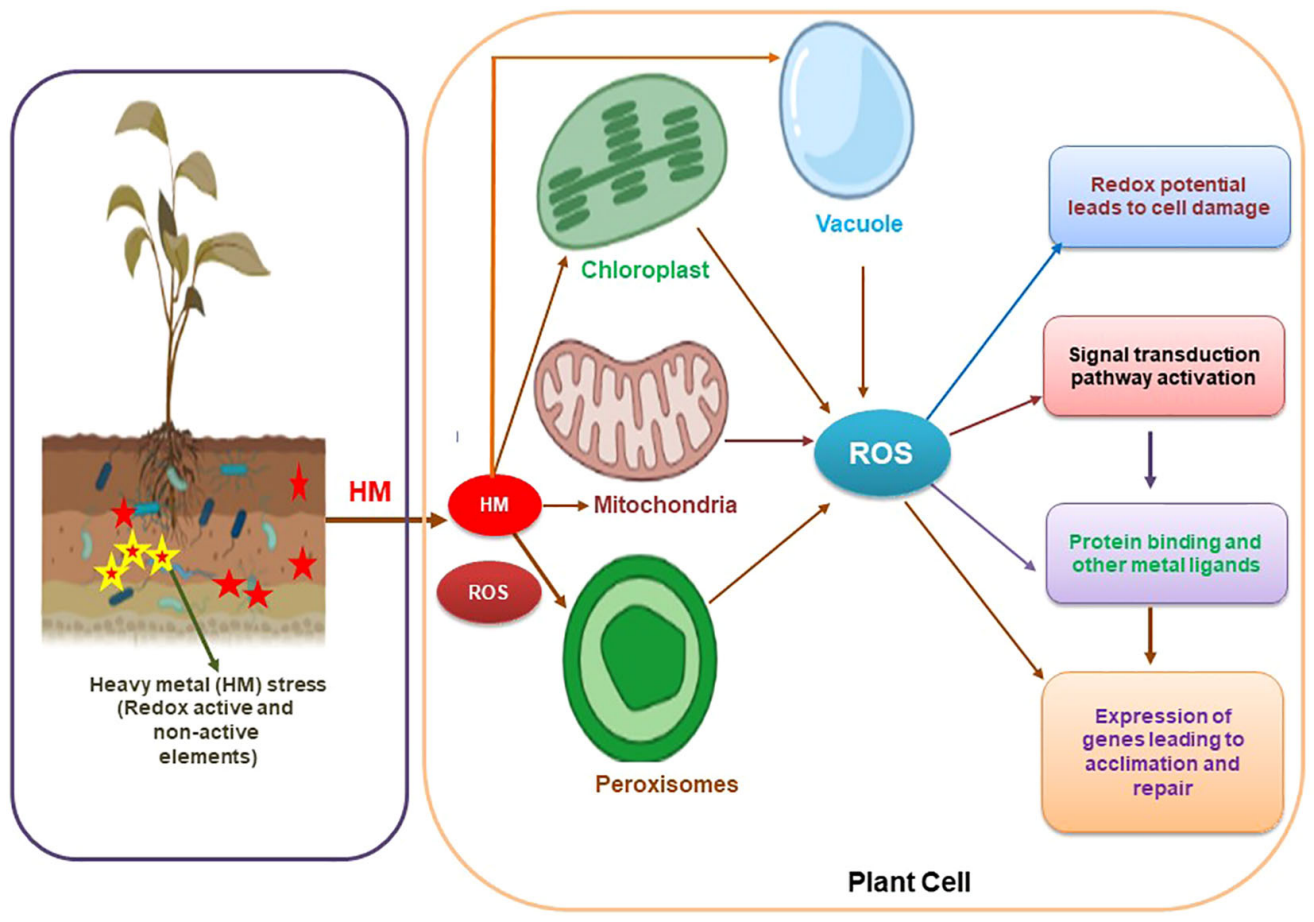
Diagrammatic representation of cellular response to heavy metal stress through the expression of genes coding for transporters and other related proteins.

Insights into the metal hyperaccumulation mechanism are deepened by conducting a comparative transcriptome analysis of many genes encoding metal ion transporters and detoxifying proteins (Wu et al., 2021). The hyperaccumulation and hypertoleranace traits are independent of genetic control and are not species-specific. Many reports depict that transporter genes are overexpressed by hyperaccumulators depending on the concentration of metal ions in diverse soil types (**Table 2**, **Figure 2**).

**Table 2:** Overexpression of transporter genes reported in common hyperaccumulators.

Species	Name of transporters	References
*Arabidopsis halleri*	*HMA1(heavy metal ATPase 1), HMA2, HMA3, HMA4 HMA5, AhHMA4, ZIP6, MATE Family-FDR3 and ZIP9, MTP1, MTP2, MTP3, MTP4, MTP5, AhMTP1, IRT3, IRT1*, *ZIP3, ZIP4, ZIP6*	(Courbot et al., 2007; Willems et al., 2007; Hanikenne et al., 2008; Assunção et al., 2010); (Lasat et al., 2000; Assunção et al., 2001; Becher et al., 2004; Dräger et al., 2004; Krämer et al., 2007; Gustin et al., 2009; Verbruggen et al., 2009b; Hanikenne and Nouet, 2011; Liu et al., 2017; Mishra et al., 2017) (Halimaa et al., 2014; Corso et al., 2018; Halimaa et al., 2019; Corso and de la Torre, 2020) (Guerinot, 2000; Schvartzman et al., 2018)
*T. caerulescens*	*HMA3, HMA4, YSL* (yellow stripe-like) family- *TcYSL3, TcYSL5 and YSL7, ZIP* (zinc-regulated transporter, iron-regulated transporter-related protein)*, ZTN1and ZTN2, MATE Family-FDR3, NRAMPs, MTP1*	(Dräger et al., 2004; Gustin et al., 2009; Assunção et al., 2010; Liu et al., 2017; Mishra et al., 2017)
*T. goesingense*	*MTPs* (metal transport proteins)-*MTP1*	(Persans et al., 2001)
*Convolvulus arvensis L*	*PIMs*	(Massa et al., 2010)
*S. plumbizincicola*	*SpHMA1, HMA3*	(Gendre et al., 2007; Liu et al., 2017; Mishra et al., 2017)
*Sedum alfredii*	*SaZIP4*	(Deng et al., 2007)
*Thlaspi arvense L.*	*ZNT1*	(Reeves and Brooks, 1983)
*Pteris vittata L.*	NIP (Nodulin 26-like Intrinsic Proteins) subfamily, *AtMTP11, CsMTP8, OsMTP8.1, ShMTP1*	(Ashraf et al., 2011; Wan et al., 2017)
*Arabidopsis lyrata*	*AhHMA4* transporter	(Merlot et al., 2018)
*N. caerulescens*	*MTP1, TcYSL3, TcHMA3, TcYSL5, TcYSL7, IRT3, IRT, ZNT1, ZNT2, NcZNT1, NcZNT2, NcZNT5*	(Pence et al., 2000; Assunção et al., 2001; Papoyan et al., 2007; Küpper and Kochian, 2010; Ueno et al., 2011; Milner et al., 2012; Halimaa et al., 2019)
*Pteris vittata L.*	*NIP* (Nodulin 26-like Intrinsic Proteins) subfamily, *AtMTP11, CsMTP8, OsMTP8.1, ShMTP1*	(Ashraf et al., 2011)
*Merwilla plumbea*	*OsNRAMP5, OsNRAMP1, TcZNT1/TcZIP4*	(Lux et al., 2011)
*N. goesingensis*	*MTP1*	(Gustin et al., 2009)

The copy number expansion of transporter genes within the genome and the strong promoter are usually activated by cis-regulatory elements resulting in the hyperexpression of genes. The central mechanism of metal ion hyperaccumulation involves many genes expressed for metal ion transporters. Therefore, it is necessary to understand the mechanism behind the transport of metal ions and trace the expression of genes to devise strategies for developing transgenic plants for heavy metal hyperaccumulation. A comparative expression analysis reported that later genomic evolution was displayed by the enhanced copy number of transporters such as; *HMA4* to 3 *and MTP1* to 5 in *Arabidopsis halleri* compared to single-copy found in wild-type *A. thaliana* (Hanikenne et al., 2008; Shahzad et al., 2010). Similar types of enhancement in expression were due to a five times more copy number of *TcHMA3* transporter to accumulate Cd in ecotypes of *N. caerulescens* (Ueno et al., 2011). Upon expression profiling of two hyperaccumulators, overexpression of genes occurred, which are involved in Zn/Cd uptake, their loading to xylem, transport, and chelation (Balafrej et al., 2020; Sharma et al., 2021a). In addition, it was observed that a higher copy number resulted in over-expression of *TcHMA3* gene encoding Cd transporters in Saint- Laurent-le-Minier (Ganges) population compared to the Prayon population of *N. caerulescens* (Ueno et al., 2011). In *A. halleri, the AhMTP1* gene encodes vacuolar membrane Zn/H^+^ antiporter, expressed 20-folds higher due to high copy number in leaves than *A. thaliana* (Becher et al., 2004; Dräger et al., 2004). Due to the higher copy number of *MTP1*, a Zn transporter is highly expressed in *A. halleri* to mediate hypertolerance (Dräger et al., 2004).

Microarray-based transcriptome analysis has led to the identification of several genes responsible for metal ion transport and their chelation in model organisms like *A. halleri* or *N. caerulescens* (Becher et al., 2004; Chiang et al., 2006; Filatov et al., 2006; Hammond et al., 2006; Talke et al., 2006; van de Mortel et al., 2006; Weber et al., 2006). Hyperaccumulators result in overexpression of transporters such as *AhHMA4*, in xylem parenchyma and pericycle of the root, and in *A. halleri* shoot tissues *viz*., cambium and xylem parenchyma (Hanikenne et al., 2008). Moreover, *MTP1* was overexpressed in *A. halleri* and *N. caerulescens* to transport Zn and Cd (Dräger et al., 2004; Gustin et al., 2009). Transport of Cd was mediated by overexpression of *HMA3* in leaf epidermal cells of *Sedum plumbizincicola.* At the same time, *HMA3* was predominantly expressed in bundle sheath and mesophyll cells in *A. halleri* and *N. caerulescens* (Liu et al., 2017: Mishra et al., 2017). The transformation of *TgMTP1* in *A. thaliana* resulted in enhanced accumulation of Zn into vacuoles, attributing to Zn tolerance (Gustin et al., 2009). Several transporters were overexpressed, such as SpHMA1 in *S. plumbizincicola*, *SaZIP4* in *Sedum alfredii*, and *TcYSL3*, *TcHMA3, TcYSL5*, and *TcYSL7* in *N. caerulescens* plants for transport of metal ions (Gendre et al., 2007; Ueno et al., 2011; Zhao et al., 2019). It is pertinent to mention that the expression of transporter genes very often varies concerning metal ion supply, tissue, organ, and populations of hyperaccumulators (Krämer et al., 2007; Verbruggen et al., 2009b; Küpper and Kochian, 2010; Visioli et al., 2014). Under sufficient Zn supply, *N. caerulescens* and *A. halleri* overexpress several transporter genes, such as *IRT3*, *IRT1*, and *ZIP* genes, for higher accumulation of metal ions (Assunção et al., 2001; Becher et al., 2004; Krämer et al., 2007; Verbruggen et al., 2009b; Hanikenne and Nouet, 2011; Halimaa et al., 2014; Corso et al., 2018; Schvartzman et al., 2018; Halimaa et al., 2019; Corso and de la Torre, 2020). In contrast, during the low availability of Zn, *ZNT1* and *ZNT2* genes encoding transporters were overexpressed in *N. caerulescens* (Pence et al., 2000; Assunção et al., 2001). Differential expressions of *NcZNT1* and *NcZNT2* transporter genes were reported in the roots and shoots of *N. caerulescens.* Moreover, tissue-specific expression was identified for expression of *NcZNT1* in stellar parenchyma cells, pericycle, and very low expression in cortex and rhizodermis in *N. caerulescens* (Milner et al., 2012).

Upon exposure to Zn deficient soil, a tissue-specific expression pattern of *NcZNT1* was observed in *N. caerulescens* (Milner et al., 2012). In addition, it is reported that *NcZNT1* was differentially expressed in shoot apical meristem and mesophyll, bundle sheath, and stomatal guard cells. On the contrary, *NcZNT5* expression was limited to young leaves, especially its epidermal cells (Küpper and Kochian, 2010; Milner et al., 2012). Expression of the *HMA4* gene occurs in roots as well as shoots of *A. halleri* and *N. caerulescens* to load and unload Cd and Zn into the xylem (Bernard et al., 2004; Papoyan and Kochian, 2004; Talke et al., 2006; van de Mortel et al., 2006; Craciun et al., 2012; Visioli et al., 2014; Mishra et al., 2017). Moreover, it is reported that *HMA4* encoding Cd and Zn metal ion transporter is overexpressed in the roots and shoots of both *A. halleri* and *T. caerulescens* metal ion hyperaccumulators (Bernard et al., 2004; van de Mortel et al., 2006; Courbot et al., 2007). The *NcZNT5* overexpression was observed in epidermal cells instead of in guard and subsidiary cells in young leaves of *N. caerulescens* (Küpper and Kochian, 2010). In contrast, an opposite expression pattern was observed in mature leaves, wherein, *NcZNT5* was overexpressed in guard cells rather than epidermal cells (Küpper and Kochian, 2010). Both these findings back up the adaptation of plants to hyperaccumulation of metal ions in correlation with the developmental stage of plants. Further, the RNA interference technique showed that higher expression of *AhHMA4* was responsible for hypertolerance to Cd and Zn metal ions in *Arabidopsis halleri* (Papoyan and Kochian, 2004). In addition, the RNAi technique showed higher transcription of *NcZNT1* (*Zn Transporter 1*) genes encoding a transporter of Cd and Zn in *N. caerulescens* (Pence et al., 2000). Under higher concentration of Zn, several genes of the ZIP family have been highly expressed in *A. halleri* and *N. caerulescens* (Assunção et al., 2001; Becher et al., 2004; Weber et al., 2004; Talke et al., 2006; van de Mortel et al., 2006; Lin et al., 2009). In *A. halleri*, several transporter genes, such as ZIP family members viz. *ZIP3, ZIP4*, and *ZIP6* are responsible for the influx of metal ions from the rhizosphere to roots and are highly expressed due to high copy numbers (Guerinot, 2000). In addition, overexpression of several *ZIP* family member transporters has been reported in *A. halleri* and *T. caerulescens* to hyperaccumulate Zn metal ions (Becher et al., 2004; Weber et al., 2004; Filatov et al., 2006; Hammond et al., 2006; Talke et al., 2006; Krämer et al., 2007). Expression analysis and RNAi-produced lines identified tissue-specific expression of *NgMTP1*encoding *MTP1* to accumulate Zn in shoots of hyperaccumulators (Desbrosses-Fonrouge et al., 2005).

Comparative analysis showed that *ZNT1* was overexpressed in roots of *T. caerulescens* in comparison to non-accumulator *T. arvense* (Pence et al., 2000). The overexpression of *ZNT1* was further confirmed by microarray analysis (Hammond et al., 2006; van de Mortel et al., 2006). Another transporter, *ZTP1*, a homolog of *AtMTP1*, was highly expressed in *T. caerulescens* to accumulate metal ions in vacuoles (Assunção et al., 2001). Two populations of *T. caerulescens* were found to differentially express two *ABC* (ATP-binding cassette) transporters in their shoots to hyperaccumulate Zn (Hassinen et al., 2007). Zn compartmentation and transport is efficiently mediated by overexpression of *HMA3* gene coding *P1B-ATPase T*. *caerulescens* and *A. halleri* (Craciun et al., 2006; van de Mortel et al., 2008). In addition, *CAX* gene encoding cation exchange mediates enhanced Cd sequestration (Craciun et al., 2006; van de Mortel et al., 2008). *FDR3* is another transporter belonging to the *MATE* (Multidrug and Toxin Efflux) gene family of transporters overexpressed in the root pericycle of *A. halleri* and *T. caerulescens* to hyperaccumulate Fe (Talke et al., 2006; van de Mortel et al., 2006). In *T. caerulescens* three genes viz. *TcYSL3, TcYSL5*, and *YSL7* are over-expressed to mediate vascular loading and transport of Ni and Fe in the form of Nicotinamide-Ni complex and Nicotainmide-Fe complex (Gendre et al., 2007). The overexpression of *NIP* genes might be responsible for transporting As from roots to the xylem vessels in *Pteris vitata* (Zhao et al., 2009). The comparative RNA-seq analysis reported that Ni transport is highly regulated by overexpression of the *ZIP* family in *S. coronatus.* Moreover, the expression pattern of *IRT1* and *ZIP10* varied between various populations of *N. caerulescens* (Corso and de la Torre, 2020). The overexpression of Ni transporter genes through high copy number and changing dynamics of promoter activity helps decode Ni’s transport mechanism by hyperaccumulators.

### 3.2 Heavy metal transporters

**Figure 3:**
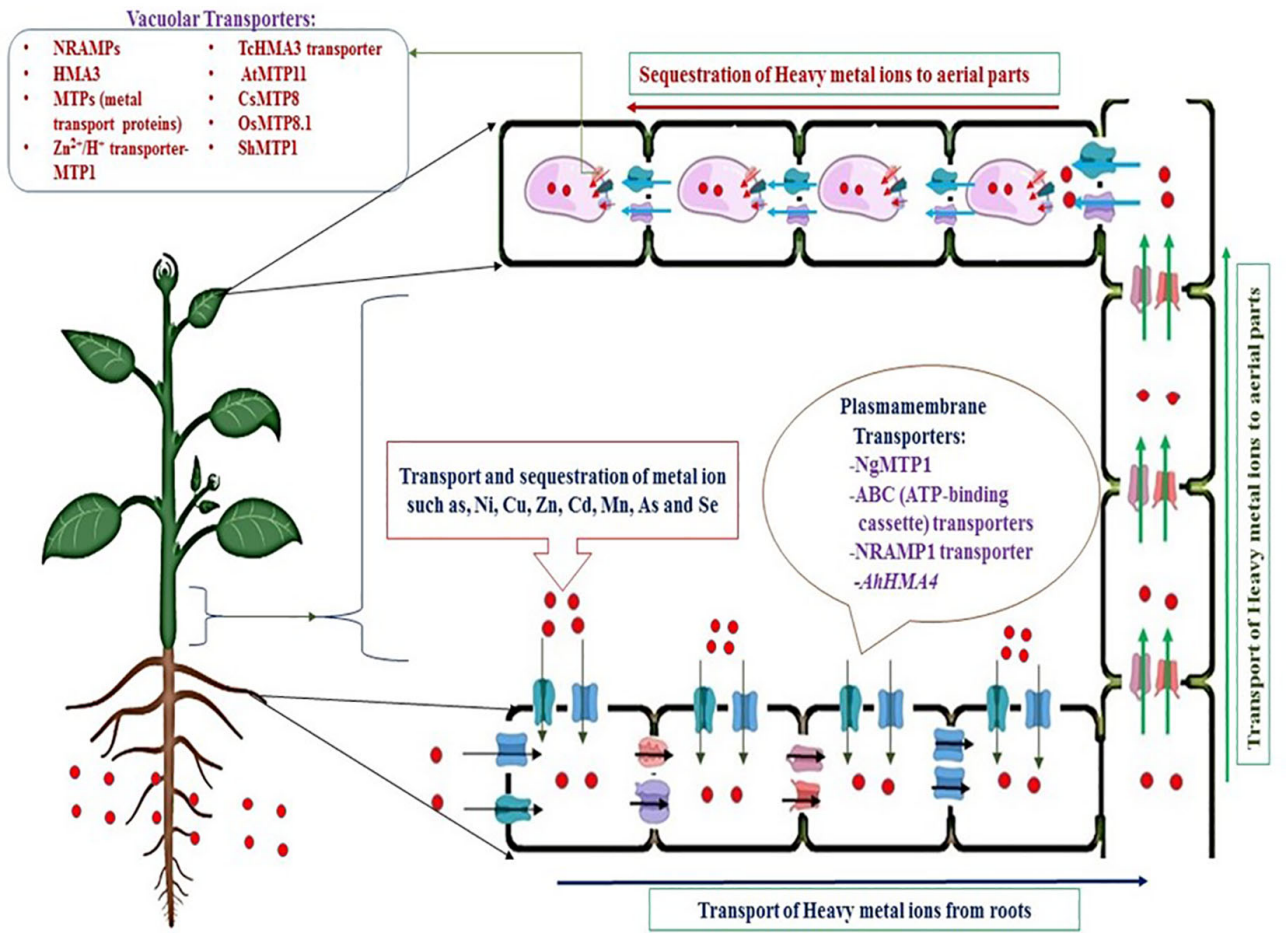
Mechanisms of transportation of heavy metals from contaminated soils through metal ion transporters by active transport.

The transport of metal ions occurs through the active accumulation of metal ions, usually generated through the air or deposited metalloids on the leaves of plants (Jogawat et al., 2021; Yang et al., 2021). In addition, inactive accumulation of metals occurs from the soil through roots and their transport through the xylem to the aerial parts of plants, such as stems, leaves, and other parts of shoots, by diverse classes of metal ion transporters (Feki et al., 2021; Jamla et al., 2021). Coefficient bioaccumulation of metal ions needs transport through pumps against the concentration gradient. Consequently, the transport of HMs from roots to aerial parts involves active transporters found in the plasma membrane and vacuolar membrane, these transporters require energy, usually in form of ATP to concentrate metal ions into storage organelles, such as vacuoles (**Figure 3**). It must be noted that vacuoles of epidermal cells store higher concentrations of metal ions as compared to other cellular organelles due presence of enzymes like proteinases, phosphatases and lipases (Feki et al., 2021).

**Figure 4:**
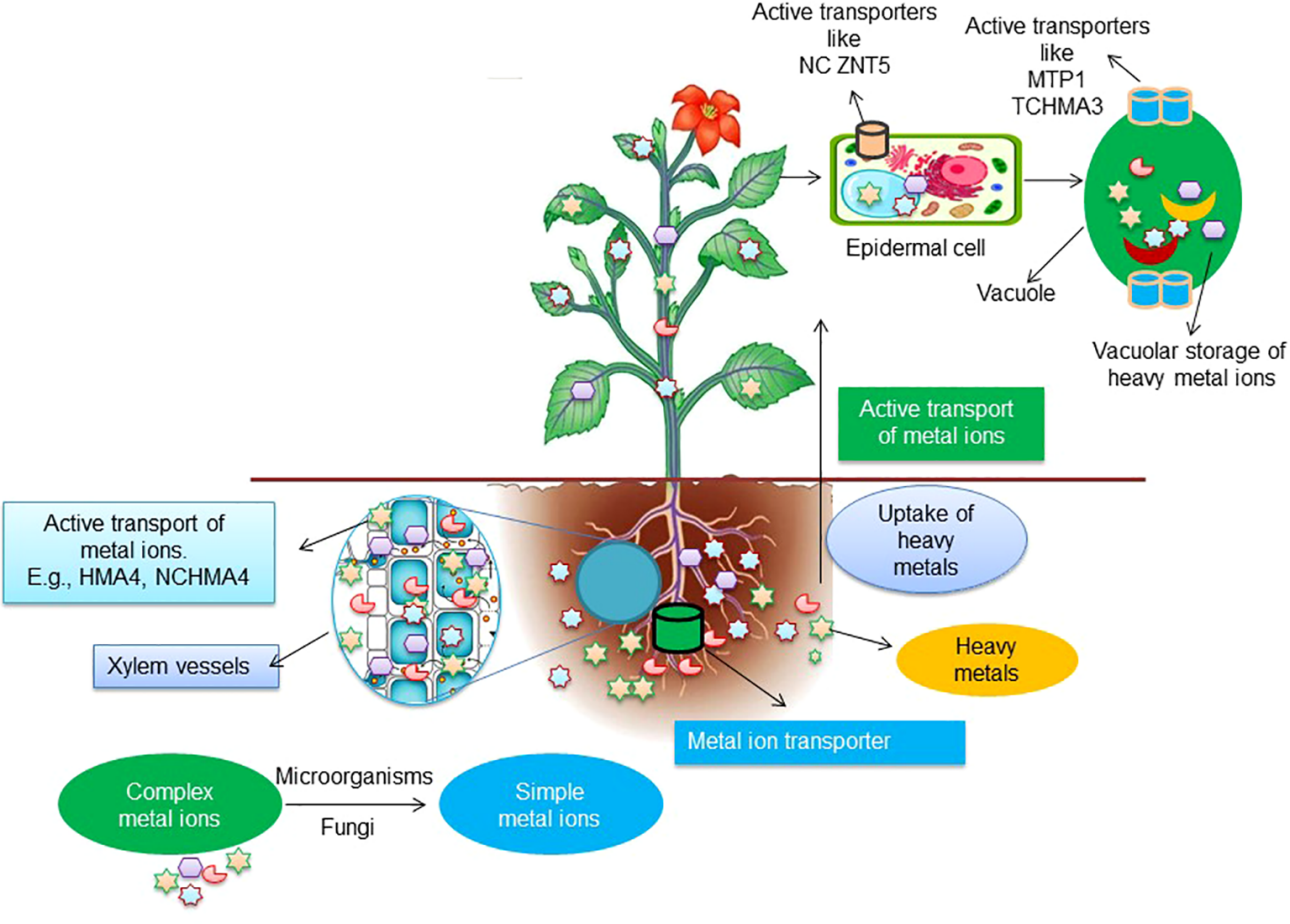
A model demonstrating the mechanism of transportation of heavy metals ions from roots to the aerial parts of plants.

On average, up to 200 times more metal ion accumulation is mediated by transporter genes overexpressed by hyperaccumulator species compared to non-hyperaccumulators (Pence et al., 2000; Assunção et al., 2001; Becher et al., 2004; Hanikenne et al., 2008; Verbruggen et al., 2009b). Transcriptome analysis identified several metal ion transporter hyperaccumulators in *Brassicaceae*. A great deal of metal ion transport has been explored through studies on *ZIP* transporters, as they are involved mainly in the uptake of metal ions (Krämer et al., 2007; Andresen et al., 2018). The hyperaccumulation of metal ions is directly related to a higher number of *ZIP* transporters in the plasma membrane of leaf epidermal cells and transpiration termination in these cells (Küpper et al., 2009; Schneider et al., 2013). In addition, (Pence et al., 2000) reported that the *ZNT1* transporter, a homolog of *AtZIP4*, transports Zn with higher affinity and Cd uptake with low affinity. The CDF (cation diffusion facilitators) family of the transporter are involved in the transport of metal ions such as Fe^2+^, Co^2+^, Zn^2+^, Mn^2+^, and Cd^2+^ from the cytoplasm to organelles like vacuoles, endoplasmic reticulum, etc. (**Figure 4**) (Peiter et al., 2007). Moreover, metal ion transport into vacuoles occurs through *CDF* (cation diffusion facilitator), *MTP* (metal tolerance protein), and *P1B* type *ATPase HMA3* (Krämer et al., 2007; Verbruggen et al., 2009b; Andresen et al., 2018). Many studies have reported on two Zn/Cd hyperaccumulator models, *T. caerulescens* and *A. halleri* (Frérot et al., 2010; Krämer, 2010). These findings strongly suggested that the differential expression and regulation of common genes rather than a novel gene set was mainly involved in the metal hyperaccumulation mechanisms (Verbruggen et al., 2009a). The cation transporters belonging to the *ZIP* family (zinc-regulated and iron-regulated transporter proteins) are primarily located at the plasma membrane of roots in both Cd/Zn hyper and non-hyperaccumulators. The constitutive overexpression of *ZIPs* genes in *T. caerulescens* (*ZTN1* and *ZTN2*) and *A. halleri* (*ZIP1* and *ZIP6*) led to enhanced uptake of Zn irrespective of the exterior Zn concentration (Assunção et al., 2010; Lira-Morales et al., 2019). While in Cd/Zn non-hyperaccumulator plants, the expression of *ZIPs* was detected only under Zn-deficient conditions, suggesting a Zn-mediated regulation unlike the constitutive expression observed in the hyperaccumulator counterpart (Weber et al., 2004; Yan et al., 2020). Although Zn transporters mediate the uptake of Cd in *A. halleri* and most ecotypes of *T. caerulescens*, Zn is preferably being transported over Cd (Zhao et al., 2002). Interestingly, (Lombi et al., 2001) reported that the Ganges ecotype of *T. caerulescens* could accumulate a very high level of Cd in their aerial parts. Cd uptake is not influenced by Zn concentration, indicating the presence of an efficient cadmium-specific transport system in the roots of this ecotype.

*ZIP* family of selective cation transporters catalyzes the transport of Ni. This family of transporters encodes *ZRT* (zinc-regulated transporter)/IRT (iron-regulated transporter) proteins consisting of 8 transmembrane domains and a metal-binding domain in the extracellular loop of the transporter (Guerinot, 2000; Kochian, 2000; Rogers et al., 2000; Potocki et al., 2013). The *IRT1* transporter uptakes metal ions via plasma membrane and through the trans-Golgi network, where they are found predominantly (Barberon et al., 2011). Even though the specificity of *IRT1* varies among species, it is versatile for the transport of a wide range of metal ions such as Co, Cd, Ni, Fe, Zn, and Mn (Rogers et al., 2000; Schaaf et al., 2006; Halimaa et al., 2014, 2019). Vert et al. (2002) reported that *IRT1* depends on the availability of the metal ion; for example, on the scarce availability of Fe, other metal ions are transported to *IRT1* found in rhizodermal root cells. The uptake of metal ions occurs through cellular metal ion transporters such as *ZIP4* for Cd, Cu, Zn, *ZIP6* for transport of Zn and Mn, and only Zn by *ZIP10/11* (Krämer et al., 2007; Corso and de la Torre, 2020). Other transporters help accumulate metal ions; for example, Cu influx occurs via *ATPase HMA5I*, and transport from apoplast to the cytosol occurs via *NRAMP1* transporter. The long-distance transport of metal ions is regulated by several transporters, such as *HMA4* (heavy metal *ATPase* 4), the *YSL* (yellow stripe-like) family, and *ZIP* (zinc-regulated transporter, iron-regulated transporter-related protein) family members (Verbruggen et al., 2009b). Moreover, other transporters involved in long-distance metal transport include *TcYSL3* and *TcYSL7* (Gendre et al., 2007). It is also reported that vacuolar sequestration of metal ions occurs through *NRAMPs* (natural resistance-associated macrophages), *HMA3*, and *MTPs* (metal transport proteins) (Verbruggen et al., 2009b). The *AhHMA4* encodes P-type *ATPases* found on the plasma membrane of hyperaccumulators and is responsible for pumping metal ions such as Zn and Cd to confer their tolerance (Talke et al., 2006; Courbot et al., 2007).

Expression analysis identified the *AhHMA4* transporter (Hanikenne et al., 2008) to mediate the xylem loading of metal ions in roots and leaves. In addition, the transport of Zn from roots and shoots was reported to be mediated by *SaZIP4* transporter *S. alfredii* (Yang et al., 2018). It was further validated by deploying RNA interference studies that Zn and Cd were highly transported by *AhHMA4* transporter (Hanikenne et al., 2008). Accumulation and sequestration of Zn metal ions was subjected to the overexpression of Zn^2+^/H^+^ transporter-*MTP1* on the vacuolar membranes (Desbrosses-Fonrouge et al., 2005; Gustin et al., 2009) as the *MTP1* found in epidermal, and mesophyll cells have been reported to be involved in the influx of Cd, Ni and Zn in the vacuoles (Küpper and Kochian, 2010; Schneider et al., 2013).

Notably, transporters differ in their mechanism of metal ion transport, such as sequestration of Cd by *TcHMA3* transporter onto foliar vacuoles and decreasing the effect of Cd on photosynthesis *SpHMA1*chloroplast Cd exporters (Ueno et al., 2011; Zhao et al., 2019). Puig (2014) reported that copper hyperaccumulation occurs through a *COPT1* transporter belonging to the *Ctrs* family on the plasma membrane. The primary expression sites of this transporter include embryos, cotyledons, root tips, trichomes, pollen grains, and guard cells (Sancenón et al., 2004). *CDF* transporters predominantly mediate the transport of metal ions into the vacuole and Golgi complex. Metal ions modulated these transporters to induce conformational changes and transport specific metal ions (Andresen et al., 2018). *MTP1* is the versatile transporter identified in the vacuolar membranes to transport Zn, in some instances Co, Cd, Fe (II), and Ni (Krämer et al., 2007; Krämer, 2010; Hanikenne and Nouet, 2011; Sharma et al., 2016). Both *A. halleri* and *N. goesingensis* possess tolerance to Zn due to the presence of *MTP1* transporter on vacuolar membranes of shoot cells (Dräger et al., 2004; Gustin et al., 2009; Meyer et al., 2010; Shahzad et al., 2010). Cd is primarily accumulated through plant roots by transporters such as *AtNRAMP6, OsNRAMP5, OsNRAMP1, AtIRT1* and *TcZNT1/TcZIP4* (Lux et al., 2011; Sasaki et al., 2012). These transporters are involved in several hyperaccumulators metal ion uptake, transport, and detoxification. The aqua glyceroporins of the *NIP* (Nodulin 26-like Intrinsic Proteins) are reported as probable transporter proteins of arsenic (As) in plants such as *P. vitata* (Ma et al., 2008; Kamiya et al., 2009). In addition, transporters, such as *AtMTP11, CsMTP8, OsMTP8.1*, and *ShMTP1*, are identified to help transport manganese into vacuoles (Chen et al., 2013; Migocka et al., 2014). Besides, plant-microbe interactions significantly enhance hyperaccumulation and metal tolerance by facilitating metal mobilization, uptake, and detoxification. Beneficial microbes, such as rhizobacteria and mycorrhizal fungi, produce siderophores, organic acids, and phytohormones that solubilize metals, making them more bioavailable for plant uptake, while also improving root growth and nutrient acquisition. Additionally, endophytic and rhizospheric microbes can sequester metals within their cells or bind them extracellularly, reducing toxicity to the plant. These interactions further induce plant stress responses, such as the upregulation of metal transporters (e.g., *ZIP, NRAMP*) and phytochelatin synthesis, enhancing metal accumulation and tolerance. Thus, symbiotic microbial communities play a crucial role in optimizing hyperaccumulator efficiency for phytoremediation.

### 3.3 Hyperaccumulators in phytoremediation: promise, pitfalls, and the path forward

Hyperaccumulators have garnered significant attention for their potential in phytoremediation due to their ability to absorb and tolerate high concentrations of HMs and other pollutants (Yang et al., 2022). However, an overly optimistic focus on their capabilities often overlooks critical limitations that hinder their practical application. One major constraint is their typically low biomass production, which limits the total quantity of contaminants that can be extracted from the soil within a given timeframe (Yang et al., 2022; Deng et al., 2024). Additionally, many hyperaccumulator species exhibit slow growth rates, further reducing their efficiency in large-scale remediation projects. These biological constraints are compounded by environmental factors, such as soil composition and climate conditions, which may restrict their adaptability to diverse contaminated sites (Sánchez-Castro et al., 2023; Aryal, 2024). Moreover, the exclusive focus on hyperaccumulation tends to disregard potential trade-offs, such as; reduced competitive ability in natural ecosystems or increased susceptibility to pests and diseases. Without addressing these limitations, the feasibility of deploying hyperaccumulators in real-world remediation scenarios remains uncertain. A more balanced assessment that acknowledges both their potential and their shortcomings is necessary to develop realistic strategies for effective phytoremediation (Skuza et al., 2022). Future research should prioritize overcoming these challenges through genetic engineering, agronomic practices, or complementary technologies to enhance their practicality and scalability.

## 4 Conclusion and future research gaps in understanding transport regulations and metal specificity

Heavy metal pollution is a global problem worsened by different anthropogenic activities. The latter reason has pushed the scientific communities to intensify research on dealing with the phytotoxicity meditated by heavy metal ions. The metal ion toxicity can be significantly dealt with by the intervention of metal ion hyperaccumulators, which can accumulate metal ions 100-folds more than non-accumulators. Hyperaccumulator plants, the versatile plant species employed for enhancing phytoextraction, phytomining, and metal ion detoxification, are great reservoirs of genes utilized to circumvent the metal ion toxicity and remediation of contaminated soils. Many hyperaccumulators have been documented, which reduce the concentration of metal ions and detoxify them to the optimum level. In conjunction with physiological and adaptive mechanisms, their molecular studies have provided deep insights into understanding the importance of hyperaccumulators as a potential remedy for obtaining contamination-free soils. For example, physiological studies carried out in different populations of *T. caerulescens* revealed differential affinities to metal ions and the mechanism of hyperaccumulation (Zhao et al., 2002; Assunção et al., 2008). High copy number and gene duplication of transporter genes such as *ZIP4/ZNT1-2* and *IRT1* in *T. caerulescens* and *HMA4*, *MTP1*, *ZIP3*, and *ZIP9* in *A. halleri* have primarily contributed to the attribute of hyperaccumulation (Hanikenne et al., 2008). Expression analysis revealed hyperaccumulators differentially express transporter genes against the metal ion concentration in contaminated soils. They respond to the higher concentration of metal ions in elevating the expression of many genes, as mentioned in the above sections of this review. Most of these genes encode transport proteins for long-distance transport and transport with cells via plasma membrane and cellular organelles for accumulation and detoxification. Consequently, it is evident from the reviewed literature that further quest is required to unravel the physiological and molecular mechanism adapted by metal ion hyperaccumulators to withstand and hyperaccumulate toxic metals from diverse soils. In addition, insight into understanding metal ion homeostasis and regulation of the metal ion concentration in cells is of prime interest.

It must be realized that there is a lack of systematic identification and screening of plants and potential hyperaccumulators (Reeves et al., 2018). The unavailability of novel molecular approaches to decipher the exact mechanism of metal ion hyperaccumulation hinders the fruitful utilization of hyperaccumulators. Unfortunately, significantly little literature has been generated on the importance of microbiomes found in the rhizosphere of hyperaccumulators augmenting the accumulation of metal ions through roots. In addition, it must be of prime interest to the scientific community to further investigate energy allocation to hyperaccumulate metal ions. Understanding genomic evolution in correlation with ecological genomics may further pave the way to broaden our understanding of the mechanism of hyperaccumulation. Strategies including a rise in biomass or the ability to absorb and sequester (HMs) are additional major areas that demand study. However, the introduction of genetically modified plants may threaten the biodiversity of a region through the formation of superweeds, mixing and outcompeting with native species, cross-pollination with other plants, and altering the environment and the sustainable conditions for biological control agents. Before their actual deployment, therefore, substantial research measuring their influence on native biodiversity and the environment should be conducted. The employment of rhizospheric bacteria to drive root proliferation, boost plant development, and increase heavy metal tolerance and plant fitness may also offer feasible alternatives. Knowledge of the exact pathways by which each heavy metal is taken up, translocated, and sequestered in plants, identification and understanding of the role of each component of the pathway, the effect/use of the metal in the metabolic processes, and the long-term effects of large-scale phytoremediation will aid in the design of ideal plant species for hyperaccumulation by genetic engineering and other mentioned methods.

Future research should focus on genetic engineering and breeding strategies to enhance hyper-accumulation traits in high-biomass plants, leveraging CRISPR-Cas9 and omics technologies to optimize metal transporter expression and detoxification pathways (Oubohssaine and Dahmani, 2024). Additionally, integrating phytoremediation with bioenergy production (e.g., using *Miscanthus* or *Helianthus annuus*) could improve economic viability (Shackirea et al., 2022). Exploring synthetic biology to design novel chelators or hyper-accumulation pathways may further revolutionize HM remediation (Rafeeq et al., 2023). Field-scale studies, long-term ecological monitoring, and policy frameworks for phytoremediation adoption are essential to translate laboratory successes into real-world applications (Tripathi et al., 2020). Ultimately, interdisciplinary approaches combining plant physiology, microbiology, and biotechnology will be crucial in addressing global HM contamination sustainably. Achieving sustainability in phytoremediation requires a multifaceted approach to minimize phytotoxicity caused by HMs while enhancing plant efficiency (Ashkanani et al., 2024). One promising strategy is the use of soil amendments, such as; biochar, compost, and chelating agents (e.g., EDTA), which can reduce HM bioavailability and mitigate plant stress (Yin et al., 2024). Additionally, microbial-assisted phytoremediation, where plant growth-promoting rhizobacteria (PGPR) and mycorrhizal fungi enhance metal uptake and tolerance, has shown significant potential (Bhat et al., 2022b). Genetic engineering and breeding of hyperaccumulator plants to improve their metal accumulation capacity and stress resilience could further optimize phytoremediation (Nurrahma et al., 2024). The circular economy approach can be integrated by utilizing hyperaccumulator biomass in metal recovery (phytomining) or bioenergy production, reducing waste and creating economic value (Mandal et al., 2024). However, careful management is needed to prevent secondary contamination from harvested biomass. Furthermore, intercropping hyperaccumulators with cash crops could provide dual benefits soil remediation and agricultural productivity while minimizing land-use conflicts (Deng et al., 2024). Besides, understanding epigenetics’s role in adapting hyperaccumulators in extreme environmental conditions needs extensive research.

Despite significant advances, key gaps remain in understanding transporter regulation and metal specificity at the molecular level, particularly in elucidating the dynamic conformational changes that govern metal selectivity and transport efficiency. The precise mechanisms by which post-translational modifications, allosteric effectors, and cellular signaling pathways modulate transporter activity are still unclear, as are the structural determinants that enable certain transporters to discriminate between chemically similar metal ions. Additionally, the interplay between metal availability, transporter expression, and cellular homeostasis in different physiological and pathological contexts requires further exploration. High-resolution structural studies under physiologically relevant conditions, combined with advanced computational and functional assays, are needed to uncover these complexities and provide a comprehensive understanding of transporter regulation and metal specificity.
